# Gastroesophageal Reflux Disease: New Insights and Treatment Approaches

**DOI:** 10.7759/cureus.67654

**Published:** 2024-08-24

**Authors:** FNU Tanvir, Gurkamal Singh Nijjar, Smriti Kaur Aulakh, Yasmeen Kaur, Sumerjit Singh, Kanwarmandeep Singh, Abhinandan Singla, Ajay Pal Singh Sandhu, Shivansh Luthra, Harman Antaal

**Affiliations:** 1 Internal Medicine, Government Medical College Amritsar, Amritsar, IND; 2 Internal Medicine, Sri Guru Ram Das University of Health Sciences and Research, Amritsar, IND; 3 Radiology, Government Medical College Amritsar, Amritsar, IND; 4 Internal Medicine, Government Medical College Patiala, Patiala, IND

**Keywords:** personalized medicine (pm), management, diagnosis, pathophysiology, gastroesophageal reflux disease (gerd)

## Abstract

Gastroesophageal reflux disease (GERD) remains a significant global health concern, with increasing prevalence and a substantial impact on quality of life. This narrative review explores recent advances in our understanding of GERD pathophysiology, diagnosis, and management. The complex interplay of factors contributing to GERD, including lower esophageal sphincter dysfunction, transient sphincter relaxations, and esophageal motility disorders, is discussed. Emerging diagnostic techniques, such as high-resolution manometry and impedance-pH monitoring, have enhanced our ability to accurately identify and characterize GERD. The review highlights the evolving landscape of GERD treatment, from conventional approaches like lifestyle modifications and proton pump inhibitors to novel strategies including potassium-competitive acid blockers, endoscopic therapies, and minimally invasive surgical techniques. The potential role of the microbiome in GERD pathogenesis and as a therapeutic target is examined. The concept of personalized medicine in GERD management is explored, considering genetic factors, biomarkers, and individual patient profiles. Complications of GERD, including erosive esophagitis, Barrett's esophagus, and esophageal adenocarcinoma, are reviewed, emphasizing the importance of early detection and appropriate management. The economic burden and impact on the quality of due to GERD are also addressed. This comprehensive review underscores the multifaceted nature of GERD and the need for a personalized, multidisciplinary approach to its management. It highlights ongoing research efforts and emerging therapies that promise to improve outcomes for GERD patients, while also identifying areas requiring further investigation to optimize diagnosis and treatment strategies.

## Introduction and background

Gastroesophageal reflux disease (GERD) is a chronic condition characterized by the retrograde flow of gastric contents into the esophagus, causing troublesome symptoms and potential complications [1]. GERD encompasses a spectrum of presentations, ranging from mild, intermittent symptoms to severe, daily episodes that significantly impact quality of life.

The global prevalence of GERD has been steadily increasing, with substantial variations across geographical regions. In Western countries, the prevalence is estimated to be 10-20% of the adult population, while in Asia, it ranges from 2.5% to 7.8% [2]. This disparity is attributed to factors such as dietary habits, obesity rates, and genetic predisposition. Recent studies suggest a rising incidence in developing countries, possibly due to the adoption of Western lifestyles and dietary patterns [3].

The impact of GERD on quality of life is substantial and multifaceted. Patients often experience physical discomfort, sleep disturbances, and limitations in daily activities. A large-scale survey found that 73% of GERD patients reported that their symptoms affected their sleep, and 63% said it impaired their productivity at work [4]. Moreover, the chronic nature of GERD can lead to psychological distress, including anxiety and depression, further compromising overall well-being.

Conventional diagnostic techniques for GERD primarily include endoscopy to visualize mucosal damage and 24-hour pH monitoring to quantify acid exposure, while symptom assessment and empiric proton pump inhibitors (PPI) trials are often used in clinical practice. Traditional therapeutic strategies encompass lifestyle modifications (such as dietary changes and weight loss), pharmacological interventions (primarily PPI and histamine 2 {H2} receptor antagonists), and surgical options like fundoplication for refractory cases.

The economic burden of GERD is considerable, encompassing direct medical costs, indirect costs due to lost productivity, and intangible costs associated with reduced quality of life. In the United States alone, the annual direct and indirect costs of GERD were estimated to exceed $9 billion, with a significant portion attributed to prescription medications and hospital admissions [5]. The long-term management of GERD, including potential complications such as Barrett's esophagus and esophageal adenocarcinoma, further contributes to healthcare expenditure.

As GERD continues to pose significant clinical and economic challenges, there is a growing need for innovative approaches to diagnosis, treatment, and management. This review aims to explore recent advances in our understanding of GERD pathophysiology, emerging diagnostic techniques, and novel therapeutic strategies that promise to improve patient outcomes and reduce the overall burden of this prevalent condition.

## Review

Pathophysiology of GERD

The pathophysiology of GERD is complex and multifactorial, involving various anatomical and functional abnormalities. Recent advances in our understanding of GERD mechanisms have shed light on the intricate interplay between different factors contributing to this condition.

**Figure 1:**
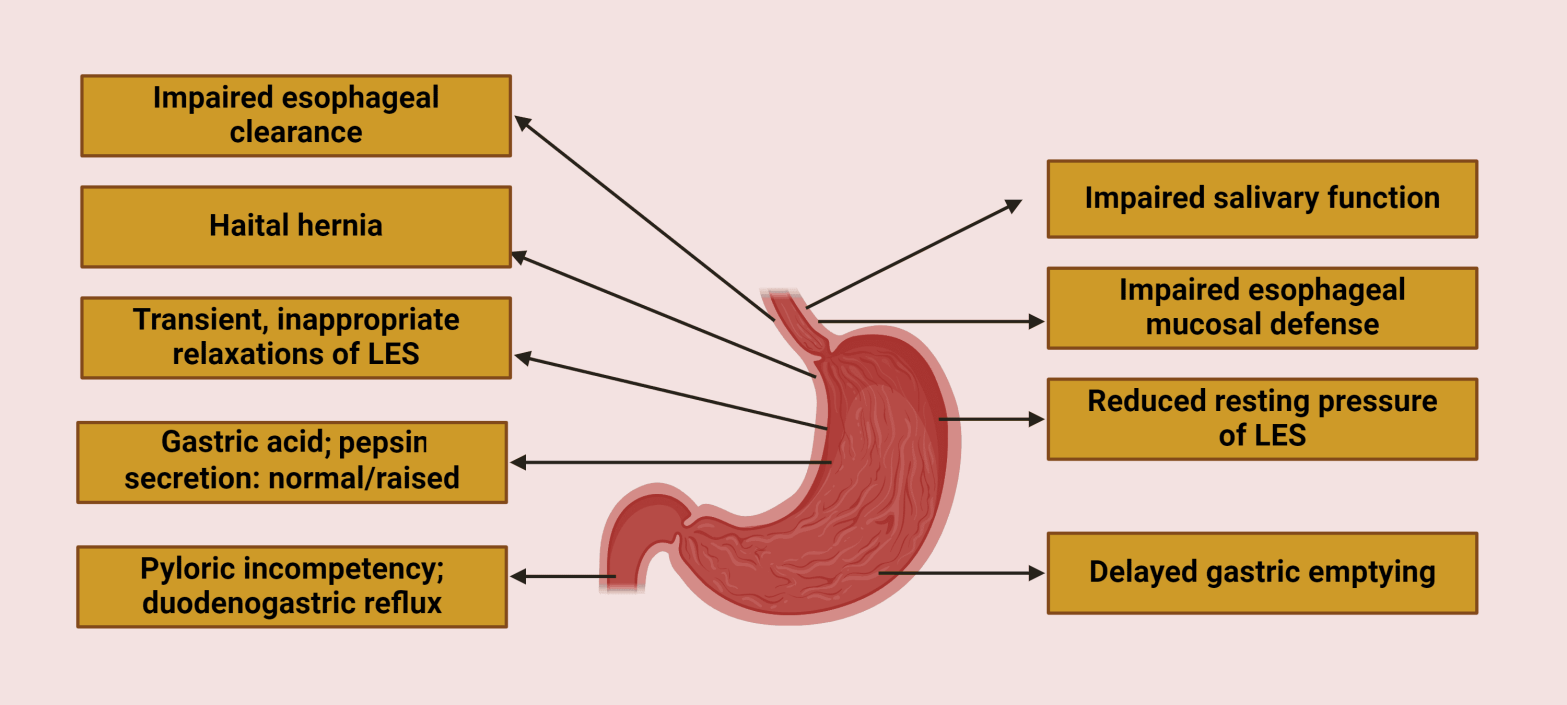
Pathophysiology of gastroesophageal reflux disease (GERD). The image is created by the authors of this study with the help of BioRender.com. LES: lower esophageal sphincter

At the core of GERD pathophysiology is the failure of the antireflux barrier, primarily composed of the lower esophageal sphincter (LES) and the crural diaphragm (Figure 1) [6]. The LES, a specialized region of circular smooth muscle at the gastroesophageal junction, plays a crucial role in preventing reflux of gastric contents into the esophagus. Recent studies have revealed that LES dysfunction in GERD patients is not solely due to reduced basal pressure but also involves impaired response to physiological stimuli such as swallowing and respiration [7].

Transient lower esophageal sphincter relaxations (TLESRs) have emerged as a key mechanism in GERD pathogenesis. These spontaneous relaxations of the LES, independent of swallowing, occur more frequently in GERD patients and are responsible for the majority of reflux episodes [8]. Recent research has elucidated the neural pathways involved in TLESRs, highlighting the role of vagal afferents and the brainstem in their regulation. The discovery of gamma-aminobutyric acid type B (GABA-B) receptor agonists as potent inhibitors of TLESRs has opened new avenues for therapeutic interventions [9].

Esophageal motility disorders also contribute significantly to GERD pathophysiology. Ineffective esophageal motility (IEM), characterized by weak peristalsis, is prevalent in up to 50% of GERD patients [10]. Recent studies using high-resolution manometry have provided deeper insights into the spectrum of GERD-related motility disorders. These include not only IEM but also fragmented peristalsis and absent contractility, which impair esophageal clearance of refluxate and prolong acid contact time [6].

The role of the esophagogastric junction (EGJ) morphology in GERD has gained increased attention. Advanced imaging techniques have revealed that EGJ disruption, including hiatal hernia, is more prevalent in GERD patients than previously recognized. The presence of a hiatal hernia not only compromises the antireflux barrier but also serves as a reservoir for refluxate, exacerbating GERD symptoms [11].

Recent research has also highlighted the importance of esophageal mucosal integrity in GERD pathophysiology. Impaired mucosal barrier function, characterized by dilated intercellular spaces and altered tight junction proteins, has been observed in GERD patients. This increased mucosal permeability may contribute to enhanced sensitivity to reflux events and the development of symptoms [12].

The concept of "volume reflux" has gained traction in recent years. Studies using impedance-pH monitoring have shown that non-acid reflux and mixed reflux events contribute significantly to symptom generation in GERD, particularly in patients with refractory symptoms on proton pump inhibitor therapy [13]. This finding underscores the importance of considering both acid and non-acid components in GERD pathophysiology and management.

Diagnosis and clinical presentation

GERD presents with a spectrum of symptoms, ranging from typical manifestations to atypical presentations that can pose diagnostic challenges. The accurate diagnosis of GERD is crucial for appropriate management and prevention of complications.

Typical symptoms of GERD include heartburn and regurgitation, which are often exacerbated by postprandial recumbency or bending over [14]. Heartburn is characterized by a burning sensation in the retrosternal area, while regurgitation involves the perception of refluxed gastric contents in the esophagus or mouth. These symptoms, when frequent and troublesome, are highly suggestive of GERD. However, the sensitivity and specificity of typical symptoms for diagnosing GERD are limited, necessitating further diagnostic evaluation in many cases [15].

Atypical symptoms of GERD encompass a wide range of manifestations, including chest pain, chronic cough, laryngitis, asthma, and dental erosions [16]. These extra-esophageal symptoms often present diagnostic challenges due to their non-specific nature and potential overlap with other conditions. The Montreal Consensus emphasized the importance of recognizing these atypical presentations and proposed a classification system that includes both esophageal and extra-esophageal syndromes associated with GERD [1].

The diagnostic criteria for GERD have evolved over time, reflecting our improved understanding of the disease. The Lyon Consensus, published in 2018, provided a comprehensive framework for GERD diagnosis, incorporating both endoscopic and physiological parameters [6]. This consensus defined conclusive evidence of GERD as the presence of Los Angeles (LA) grade C or D esophagitis, Barrett's esophagus, or peptic strictures on endoscopy, or abnormal esophageal acid exposure time on pH monitoring.

**Figure 2:**
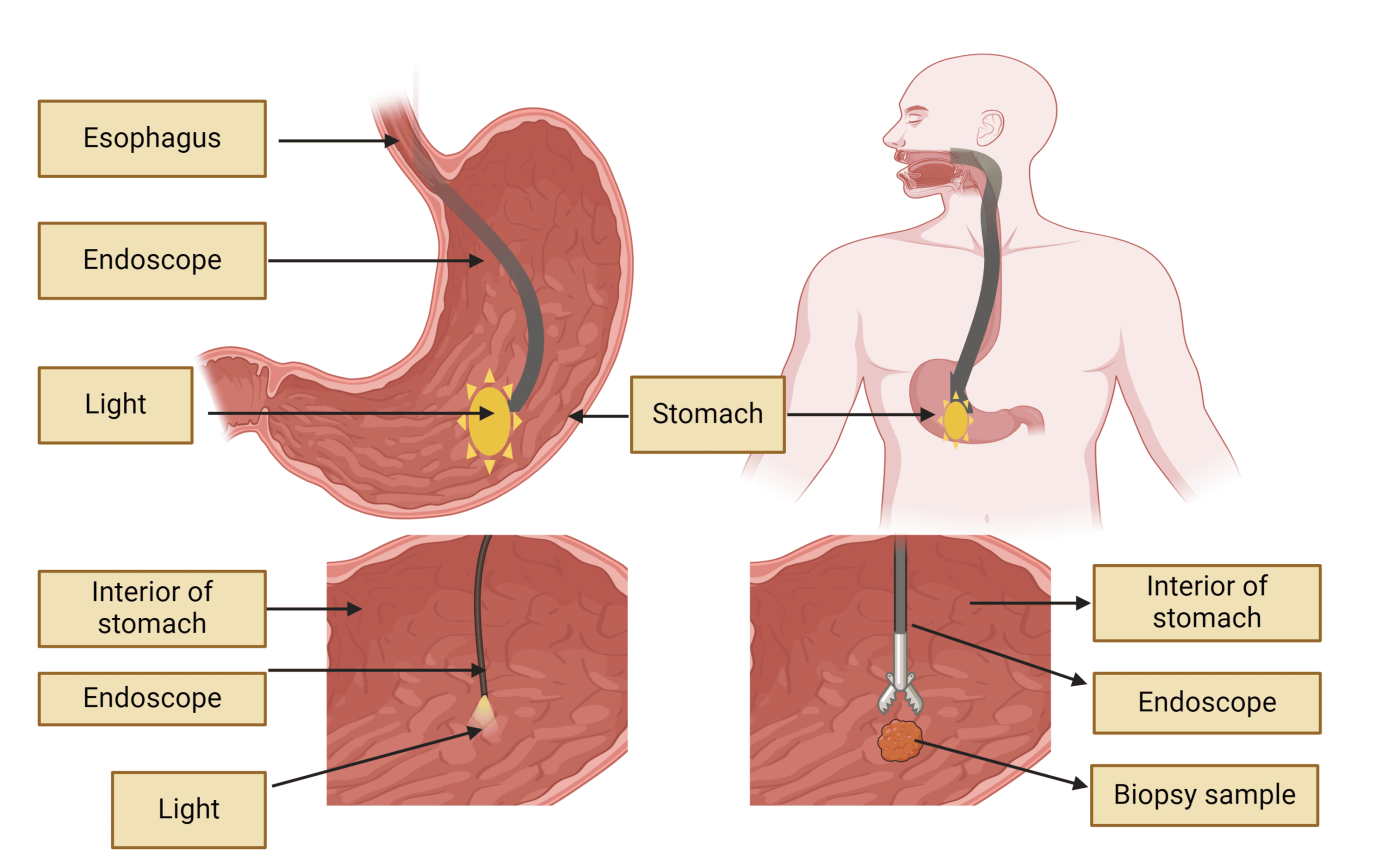
Esophagogastroduodenoscopy (EGD or upper endoscopy). The image is created by the authors of this study with the help of BioRender.com. Endoscopy (with biopsy if needed) in patients with alarm signs/symptoms, those who fail a medication trial, and those who require long-term therapy. The absence of endoscopic features does not exclude a GERD diagnosis. GERD: gastroesophageal reflux disease

One of the primary challenges in GERD diagnosis is the lack of a single, definitive test. Endoscopy, while useful for identifying complications such as esophagitis or Barrett's esophagus, has limited sensitivity for GERD diagnosis, as up to 70% of patients with typical GERD symptoms have normal endoscopic findings (Figure 2) [17]. Ambulatory pH monitoring, considered the gold standard for quantifying esophageal acid exposure, also has limitations, including day-to-day variability and the potential for false-negative results in patients with non-acid reflux [18].

Recent advances in diagnostic techniques have improved our ability to accurately identify and characterize GERD (Table 1). High-resolution manometry (HRM) has revolutionized the assessment of esophageal motility and lower esophageal sphincter function, providing detailed information on pressure topography and identifying subtle motor abnormalities associated with GERD [19]. Combined multichannel intraluminal impedance-pH monitoring (MII-pH) allows for the detection of both acid and non-acid reflux events, offering a more comprehensive evaluation of reflux patterns and symptom associations [20].

**Table 1:** Recent advances in diagnostic tools for GERD. GERD: gastroesophageal reflux disease Bravo (Shoreview, MN: Medtronic)

Technology	Advantages	Limitations
High-resolution manometry (HRM) [19]	Provides detailed pressure topography of the esophagus, helps identify motility disorders, and is useful in pre-operative evaluation.	Cannot directly detect reflux events, limited information on bolus transit, and requires specialized interpretation.
Combined multichannel intraluminal impedance-pH monitoring (MII-pH) [20]	Detects both acid and non-acid reflux, provides information on proximal extent of reflux, and correlates symptoms with reflux events.	Requires 24-hour catheter placement, patient discomfort, and may alter normal eating and activity patterns.
Wireless pH capsule (Bravo) [21]	Extended monitoring period (up to 96 hours), more comfortable for patients, and allows normal daily activities.	Only measures acid reflux, risk of capsule detachment or retention, and cannot measure impedance.
Mucosal impedance testing [22]	Rapid assessment of mucosal integrity, can be performed during endoscopy, and helps differentiate GERD from functional heartburn.	Limited long-term data, not widely available, requires specialized equipment.
Functional lumen imaging probe (FLIP) technology [23]	Assesses esophageal distensibility, useful in evaluation of eosinophilic esophagitis and achalasia, and can be performed during endoscopy.	Limited direct application to GERD diagnosis, requires specialized equipment and expertise, still being evaluated for routine clinical use.

Novel technologies are emerging to address the limitations of conventional diagnostic methods. The wireless pH capsule (Bravo; Shoreview, MN: Medtronic) provides extended pH monitoring, potentially increasing the diagnostic yield and patient tolerability compared to traditional catheter-based systems [21]. The Bravo is a catheter-free technique that utilizes a small capsule attached to the esophageal mucosa to measure acid exposure over an extended period, typically 48-96 hours. This wireless approach allows for a more comfortable and natural diagnostic experience for patients, potentially improving the accuracy of GERD diagnosis by capturing data during normal daily activities and sleep patterns. Mucosal impedance testing, which measures changes in esophageal mucosal integrity, has shown promise as a minimally invasive tool for GERD diagnosis, with high sensitivity and specificity reported in initial studies [22].

Functional lumen imaging probe (FLIP) technology offers insights into esophagogastric junction distensibility and compliance, parameters that may be relevant in GERD pathophysiology and diagnosis [23]. Additionally, molecular biomarkers, such as pepsin and bile acids in saliva or exhaled breath condensate, are being investigated as potential non-invasive diagnostic tools for GERD [24].

Complications of GERD

Erosive Esophagitis

**Figure 3:**
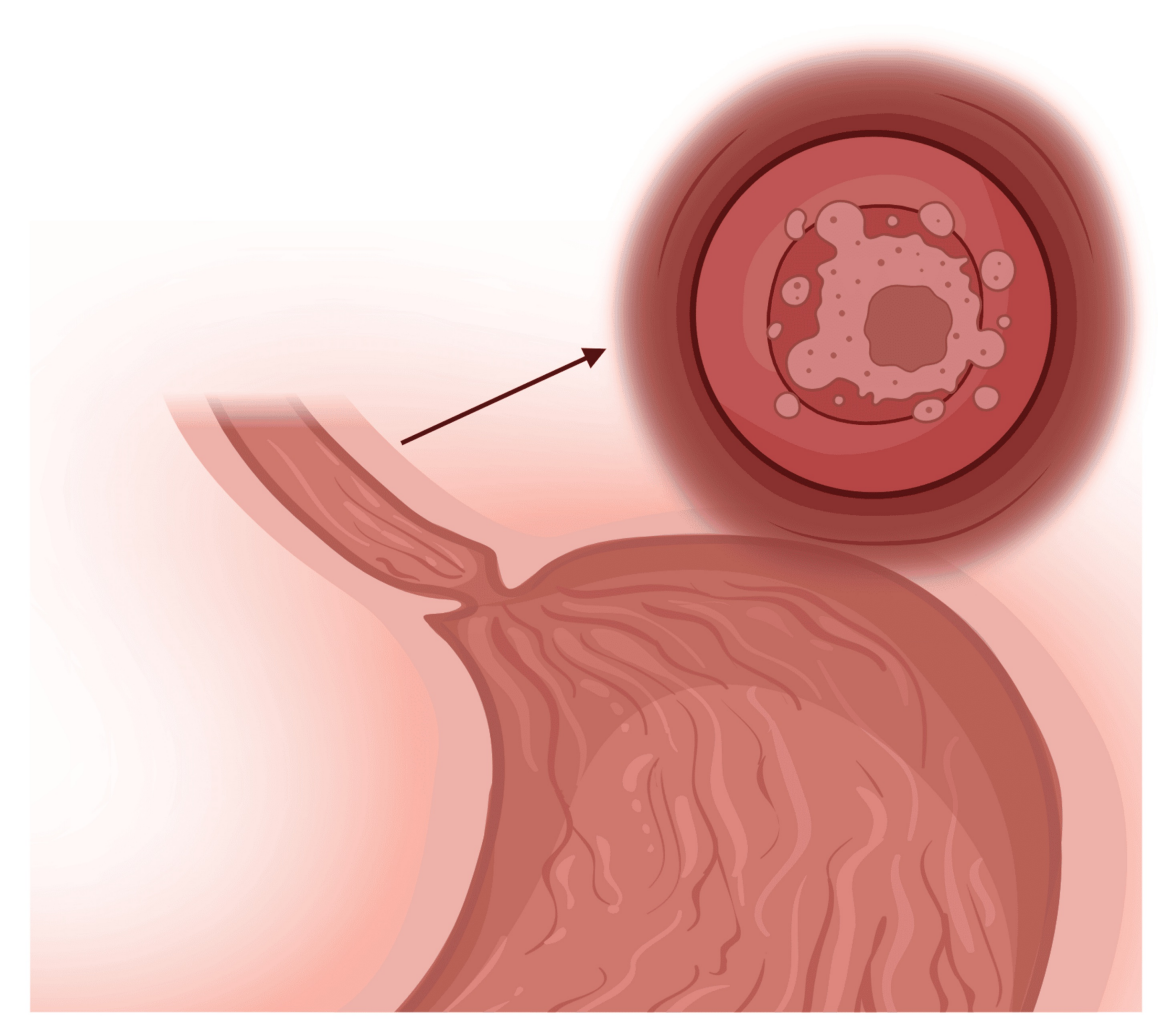
Erosive esophagitis showing inflamed, eroded areas of the esophageal lining with visible ulcerations. The image is created by the authors of this study with the help of BioRender.com.

Erosive esophagitis is a common complication of gastroesophageal reflux disease (GERD), characterized by inflammation and erosion of the esophageal mucosa (Figure 3). The Los Angeles Classification system grades the severity of erosive esophagitis from A to D based on the extent and distribution of mucosal breaks [25]. Prolonged exposure to acidic refluxate leads to epithelial damage, inflammatory cell infiltration, and potential ulceration. The prevalence of erosive esophagitis in GERD patients varies widely, with estimates ranging from 30% to 70% [26]. Risk factors for developing erosive esophagitis include increased frequency and duration of reflux episodes, presence of hiatal hernia, and impaired esophageal clearance [27]. Complications of severe erosive esophagitis include bleeding, stricture formation, and rarely, perforation. Management typically involves proton pump inhibitors (PPIs) to suppress acid production and promote mucosal healing. Endoscopic follow-up is recommended to assess healing and exclude the development of Barrett's esophagus. Recent studies have explored the role of mucosal protective agents and potassium-competitive acid blockers as alternative or adjunctive therapies for erosive esophagitis [28].

Barrett's Esophagus

**Figure 4:**
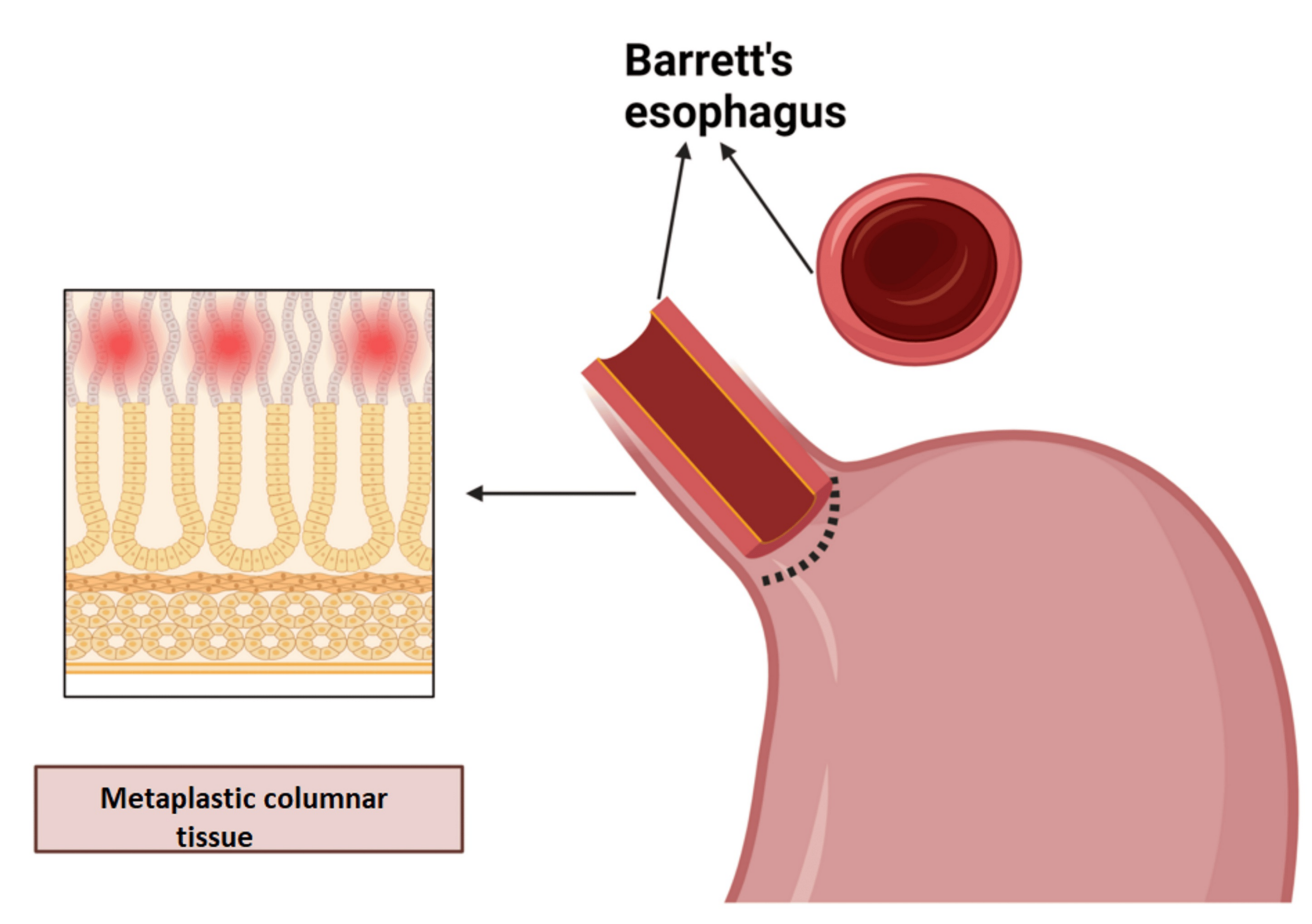
Barrett’s esophagus with metaplastic columnar epithelium replacing the normal stratified squamous lining. The image is created by the authors of this study with the help of BioRender.com.

Barrett's esophagus (BE) is a premalignant condition characterized by the replacement of normal squamous epithelium with specialized intestinal metaplasia in the distal esophagus (Figure 4). It is a significant complication of long-standing GERD, with an estimated prevalence of 5-15% among GERD patients [29]. The exact pathogenesis of BE remains unclear, but chronic exposure to acidic and biliary reflux is thought to play a crucial role in initiating the metaplastic process. Risk factors for BE include male gender, obesity, smoking, and prolonged GERD symptoms [30]. The primary clinical significance of BE lies in its association with an increased risk of esophageal adenocarcinoma, with an estimated annual conversion rate of 0.1-0.3% [31]. Endoscopic surveillance is recommended for patients with BE to detect dysplasia or early-stage cancer, although the optimal surveillance intervals remain debated. Management strategies for BE include acid suppression therapy, endoscopic eradication techniques such as radiofrequency ablation and endoscopic mucosal resection for dysplastic BE, and in some cases, esophagectomy for high-grade dysplasia or early adenocarcinoma [32]. Recent advances in biomarker research and imaging technologies, such as wide-area transepithelial sampling and confocal laser endomicroscopy, hold promise for improving risk stratification and early detection of neoplastic progression in BE patients.

Esophageal Adenocarcinoma

Esophageal adenocarcinoma (EAC) represents a severe complication of long-standing gastroesophageal reflux disease (GERD) and Barrett's esophagus (BE). The incidence of EAC has risen dramatically in Western countries over the past four decades, with a six-fold increase observed in the United States [33]. This alarming trend is attributed to the increasing prevalence of GERD, obesity, and other risk factors. The progression from GERD to BE and ultimately to EAC follows a well-established sequence of metaplasia-dysplasia-carcinoma, although the exact molecular mechanisms driving this progression are still being elucidated [34]. Risk factors for EAC include chronic GERD, BE, obesity, male gender, smoking, and certain dietary patterns. The prognosis for EAC remains poor, with a five-year survival rate of approximately 20%, largely due to late-stage diagnosis in many cases [35]. Early detection through endoscopic surveillance of high-risk individuals, particularly those with BE, is crucial for improving outcomes. Treatment strategies for EAC depend on the stage at diagnosis and may include endoscopic resection for early-stage disease, neoadjuvant chemoradiotherapy followed by esophagectomy for locally advanced disease, and palliative chemotherapy for metastatic disease [36]. Emerging therapies, including immunotherapy and targeted molecular agents, are being investigated to improve outcomes in EAC patients.

Extra-Esophageal Manifestations

Gastroesophageal reflux disease (GERD) can manifest with a variety of extra-esophageal symptoms and complications, often referred to as atypical or laryngopharyngeal reflux. These manifestations can affect the respiratory system, oral cavity, and other organs, presenting diagnostic and therapeutic challenges. Common extra-esophageal manifestations include chronic cough, asthma, laryngitis, and dental erosions [16]. The pathophysiology of these complications involves direct damage from refluxate and vagally mediated reflexes. Chronic cough associated with GERD may result from microaspiration or esophago-bronchial reflex mechanisms, while asthma can be exacerbated by reflux-induced bronchoconstriction [37]. Laryngeal manifestations, including hoarseness, globus sensation, and chronic laryngitis, are thought to result from direct contact of refluxate with laryngeal tissues [38]. Dental erosions occur due to the repeated exposure of teeth to acidic refluxate. Diagnosing extra-esophageal GERD can be challenging, often requiring a combination of symptom assessment, endoscopy, pH monitoring, and empiric treatment trials. Management typically involves aggressive acid suppression, lifestyle modifications, and in some cases, anti-reflux surgery. Recent research has focused on improving diagnostic accuracy for extra-esophageal GERD and developing targeted therapies for specific manifestations [39].

Conventional treatment approaches

Lifestyle Modifications

Lifestyle modifications are often the first-line approach in managing gastroesophageal reflux disease (GERD). These interventions aim to reduce the frequency and severity of reflux episodes by addressing modifiable risk factors. Key recommendations include weight loss for overweight or obese individuals, as excess abdominal fat increases intra-abdominal pressure and promotes reflux [40]. Dietary modifications involve avoiding trigger foods such as caffeine, chocolate, and spicy or fatty foods, as well as reducing portion sizes to decrease gastric distension [41]. Elevating the head of the bed and avoiding late-night meals can help minimize nocturnal reflux. Smoking cessation is strongly advised, as nicotine relaxes the lower esophageal sphincter. Limiting alcohol consumption and avoiding tight-fitting clothing are also recommended. While the efficacy of individual lifestyle modifications varies, a comprehensive approach combining multiple strategies has shown significant improvement in GERD symptoms and quality of life [42]. Patient education and adherence to these modifications are crucial for their long-term success in GERD management.

Pharmacological Interventions

Pharmacological interventions play a central role in GERD management, with proton pump inhibitors (PPIs) being the mainstay of treatment. PPIs effectively suppress gastric acid secretion and promote healing of erosive esophagitis, offering superior efficacy compared to other acid-suppressive medications [43]. Common PPIs include omeprazole, esomeprazole, and pantoprazole, typically administered once daily before the first meal. H2-receptor antagonists (H2RAs), such as ranitidine and famotidine, provide an alternative for mild GERD or as add-on therapy for nighttime reflux symptoms [44]. Antacids and alginates offer rapid but short-term relief for occasional symptoms. Prokinetics, like metoclopramide, may be used to enhance gastric emptying and lower esophageal sphincter pressure, although their efficacy is limited. Recent innovations include potassium-competitive acid blockers (P-CABs), which offer rapid and potent acid suppression [45]. While generally safe, long-term PPI use has been associated with potential risks, including nutrient deficiencies and increased susceptibility to certain infections, necessitating careful consideration of benefits and risks in chronic GERD management.

Surgical Options (Fundoplication)

Surgical intervention, primarily laparoscopic Nissen fundoplication, is considered for patients with refractory GERD symptoms, those who desire to discontinue long-term medical therapy, or those with complications such as large hiatal hernias [46]. The procedure involves wrapping the upper portion of the stomach around the lower esophagus to reinforce the lower esophageal sphincter and prevent reflux. Fundoplication has shown high success rates in controlling GERD symptoms, with up to 90% of patients reporting long-term satisfaction [47]. However, potential complications include dysphagia, gas bloat syndrome, and recurrence of symptoms over time. Patient selection is crucial, with preoperative evaluation including endoscopy, manometry, and pH monitoring to confirm GERD diagnosis and assess esophageal function. Alternative surgical approaches, such as magnetic sphincter augmentation (LINX device; Shoreview, MN: Torax Medical), have emerged as less invasive options with promising short-term results [48]. While surgery offers a potential long-term solution for GERD, it requires careful consideration of individual patient factors, surgical expertise, and potential risks and benefits.

Emerging treatment strategies

While traditional management approaches, such as lifestyle modifications and proton pump inhibitors (PPIs), remain the cornerstone of treatment, there is a growing interest in novel therapeutic strategies. These emerging treatments aim to address the limitations of current therapies and provide alternative options for patients who do not respond adequately to conventional treatments. The following table summarizes some of the most promising emerging treatment strategies for GERD, categorized into the following three main areas: novel pharmacological agents, endoscopic therapies, and minimally invasive surgical techniques (Table 2). Each approach not only offers unique advantages but also comes with its own set of limitations, highlighting the complex nature of GERD management and the ongoing need for research and innovation in this field.

**Table 2:** Emerging treatment strategies for gastroesophageal reflux disease (GERD).

Emerging treatment	Name of intervention	Advantages	Limitations
Novel pharmacological agents	1. Potassium-competitive acid blockers (P-CABs). E.g., vonoprazan. 2. Reflux inhibitors. E.g., lesogaberan (GABA-B agonist). 3. Prucalopride (5-HT4 agonist)	1. Rapid onset of action, more potent acid suppression than PPIs. 2. Reduces transient lower esophageal sphincter relaxations. 3. Enhances esophageal and gastric motility	1. Limited long-term safety data, potential for drug interactions [49,50]. 2. Limited efficacy in clinical trials, development halted for some agents [51,9]. 3. Primarily studied for constipation, limited GERD-specific data [52]
Endoscopic therapies	1. Transoral incisionless fundoplication (TIF). 2. Strata procedure (radiofrequency energy delivery). 3. Medigus Ultrasonic Surgical Endostapler (MUSE)	1. Less invasive than surgery, preserves anatomical structures. 2. Outpatient procedure, may reduce PPI dependence. 3. Creates endoscopic fundoplication, minimally invasive	1. Variable long-term efficacy, limited to specific patient populations [53]. 2. Conflicting evidence on long-term efficacy, potential for stricture formation [54]. 3. Limited long-term data, technical challenges in some patients [55]
Minimally invasive surgical techniques	1. Magnetic sphincter augmentation (LINX device). 2. Electrical stimulation therapy (EndoStim). 3. Single-port laparoscopic fundoplication	1. Preserves gastric anatomy, reversible procedure. 2. Augments lower esophageal sphincter function. 3. Improved cosmesis, potentially faster recovery	1. Potential for dysphagia, device erosion, limited MRI compatibility [56]. 2. Invasive procedure, limited long-term data, ongoing clinical trials [57]. 3. Technical challenges, may not be suitable for all patients [58]

Personalized medicine in GERD management

The advent of personalized medicine has opened new avenues for tailoring GERD management to individual patients, considering genetic factors, biomarkers, and unique patient profiles.

Genetic Factors Influencing GERD

Recent studies have identified several genetic variations associated with GERD susceptibility and treatment response. Polymorphisms in genes encoding for cytochrome P450 enzymes, particularly CYP2C19, have been shown to influence the efficacy of proton pump inhibitors (PPIs) [57]. Individuals with rapid metabolizer genotypes may require higher doses or alternative treatments. Additionally, variations in the gene encoding for IL-1β, a pro-inflammatory cytokine, have been linked to increased GERD risk and severity [58].

Biomarkers for Diagnosis and Treatment Response

Emerging biomarkers are enhancing GERD diagnosis and predicting treatment outcomes. Pepsin detection in saliva or exhaled breath condensate has shown promise as a non-invasive diagnostic tool for GERD [59]. Esophageal mucosal impedance measurements can differentiate GERD from functional heartburn, potentially reducing unnecessary PPI use [60]. Serum gastrin levels and histamine measurements in esophageal biopsies may predict response to acid suppression therapy [61].

Tailoring Treatment to Individual Patient Profiles

Personalized GERD management involves considering multiple factors beyond symptoms alone. Body mass index, dietary habits, and concomitant medications all influence treatment decisions [6]. For obese patients, weight loss programs may be prioritized alongside pharmacological interventions. Patients with nocturnal GERD symptoms might benefit from evening-dosed PPIs or specialized pillow systems [44].

The presence of extra-esophageal symptoms, such as chronic cough or laryngitis, may necessitate more aggressive acid suppression or consideration of surgical options [40]. In patients with refractory GERD, functional esophageal testing, including pH-impedance monitoring and high-resolution manometry, can guide therapy selection between escalated medical management and anti-reflux surgery [19].

For patients with Barrett's esophagus, a precancerous condition associated with GERD, personalized surveillance and treatment protocols based on genetic markers and endoscopic findings are being developed [32]. This approach aims to stratify patients according to their risk of progression to esophageal adenocarcinoma, potentially allowing for more targeted interventions.

As our understanding of GERD pathophysiology and individual variability grows, the future of GERD management lies in integrating genetic, molecular, and clinical data to provide truly personalized care. This approach promises to improve treatment efficacy, reduce unnecessary interventions, and enhance patient outcomes in GERD management.

Role of the microbiome in GERD

Recent research has highlighted the significant role of the microbiome in GERD pathogenesis and potential therapeutic interventions.

Esophageal and Gut Microbiome Alterations in GERD

Studies have revealed distinct differences in the esophageal microbiome between healthy individuals and those with GERD. GERD patients show a shift from Gram-positive aerobic bacteria to Gram-negative anaerobes [62]. This dysbiosis is characterized by an increase in Veillonella, Prevotella, and Fusobacterium species, and a decrease in Streptococcus [63]. The altered microbiome may contribute to esophageal inflammation and mucosal damage.

Gut microbiome alterations in GERD patients include reduced diversity and an increase in potentially pathogenic bacteria [64]. These changes may affect intestinal permeability, leading to increased acid reflux and GERD symptoms. Additionally, *Helicobacter pylori* infection, which alters gastric microbiota, has been associated with both increased and decreased risk of GERD, depending on the strain and host factors [65].

Potential Therapeutic Interventions Targeting the Microbiome

Emerging evidence suggests that modulating the microbiome could be a promising approach to GERD management. Probiotics have shown potential in alleviating GERD symptoms by improving esophageal motility and reducing inflammation [66]. Lactobacillus and Bifidobacterium strains have demonstrated particular efficacy in reducing reflux episodes and improving quality of life in GERD patients [67].

Prebiotics and synbiotics may also play a role in restoring microbial balance and reducing GERD symptoms [68]. Fecal microbiota transplantation (FMT) is being explored as a potential treatment for refractory GERD, although more research is needed to establish its efficacy and safety [69].

Understanding the complex interactions between the host and microbiome in GERD pathogenesis opens up new avenues for diagnosis and treatment. Future research should focus on developing targeted microbiome-based therapies and identifying specific microbial signatures that could serve as biomarkers for GERD progression and treatment response [70]. Table 3 summarizes the research on GERD focusing on potential new targets for therapy and advancements in diagnostic technologies.

**Table 3:** Potential new targets for therapy and advancements in diagnostic technologies in GERD. LESS GERD trial: Laparoscopic Endoscopic Single-Site Gastroesophageal Reflux Disease trial

Study/trial name or number	Intervention	Outcome
LESS GERD trial: NCT04001777 [71]	Vonoprazan in erosive esophagitis.	Study ongoing, results not yet available
Phase III randomized trial [72]	Vonoprazan vs. lansoprazole in Asian patients with erosive esophagitis	Vonoprazan showed superior efficacy in healing erosive esophagitis compared to lansoprazole
CALIBER trial: NCT02505945 [73]	Prucalopride in GERD patients	Study completed; results not yet available
Retrospective cohort study [74]	Transoral incisionless fundoplication (TIF)	TIF showed long-term effectiveness in selected patients with GERD
Expert review [75]	Functional lumen imaging probe (FLIP)	FLIP is recommended as a useful tool for the management of esophageal disorders
Pharmacologic review [76]	Various pharmacologic treatments for esophageal disorders	Review of mechanisms and efficacy of different drug classes for esophageal disorders
Complications review [77]	Anti-reflux surgery	Overview of potential complications following anti-reflux surgery
Clinical practice study [78]	Wireless pH capsule	Wireless pH monitoring showed clinical utility in GERD diagnosis and management
Randomized controlled studies [79]	Dexlansoprazole MR in erosive esophagitis	Dexlansoprazole MR demonstrated efficacy in healing erosive esophagitis

## Conclusions

Based on the comprehensive review presented, it is evident that our understanding of GERD has significantly advanced in recent years. The multifaceted nature of GERD pathophysiology, involving complex interactions between anatomical, functional, and molecular factors, underscores the need for personalized approaches to diagnosis and treatment. Emerging diagnostic technologies, such as high-resolution manometry and impedance-pH monitoring, are enhancing our ability to accurately identify and characterize GERD. Novel therapeutic strategies, including potassium-competitive acid blockers, endoscopic therapies, and minimally invasive surgical techniques, offer promising alternatives for patients who do not respond adequately to conventional treatments. The growing recognition of the role of the microbiome in GERD pathogenesis opens up new avenues for targeted interventions. As we move towards personalized medicine in GERD management, integrating genetic, molecular, and clinical data will be crucial for optimizing patient outcomes. Future research should focus on refining these emerging approaches, developing biomarkers for risk stratification and treatment response, and addressing the long-term safety and efficacy of novel interventions. Ultimately, a multidisciplinary approach that considers the individual patient's risk factors, symptoms, and preferences will be key to improving the management of this prevalent and impactful condition.
